# What goes up must come down: dynamics of type 1 interferon signaling across the lifespan

**DOI:** 10.3389/fimmu.2025.1654604

**Published:** 2025-10-16

**Authors:** Lucy Hartnell, Patricia Agudelo-Romero, Samuel T. Montgomery, Rym Ben-Othman, Valerie Verhasselt, Stephen M. Stick, Tobias R. Kollmann

**Affiliations:** ^1^ Medical School, The University of Western Australia, Nedlands, WA, Australia; ^2^ Wal-yan Respiratory Research Centre, The Kids Research Institute Australia, Perth, WA, Australia; ^3^ Australian Research Council Centre of Excellence in Plant Energy Biology, School of Molecular Sciences, The University of Western Australia, Perth, WA, Australia; ^4^ European Virus Bioinformatics Center, Friedrich-Schiller-Universitat Jena, Jena, Germany; ^5^ Center for Child Health Research, The University of Western Australia, Nedlands, WA, Australia; ^6^ RAN-Biolinks Canada Ltd., Toronto, ON, Canada; ^7^ Larsson-Rosenquist Foundation Centre for Immunology and Breastfeeding, School of Medicine, The University of Western Australia, Perth, WA, Australia; ^8^ Immunology and Breastfeeding Team, The Kids Research Institute Australia, Perth, WA, Australia; ^9^ Department of Pediatrics, Centre for Child Health Research, The University of Western Australia, Nedlands, WA, Australia; ^10^ Department of Respiratory and Sleep Medicine, Perth Children’s Hospital, Perth, WA, Australia; ^11^ Canadian Center for Vaccinology, Dalhousie University, IWK Health Centre and the Nova Scotia Health Authority, Halifax, NS, Canada; ^12^ Department of Pediatrics, Dalhousie University, Halifax, NS, Canada; ^13^ Department of Microbiology and Immunology, Dalhousie University, Halifax, NS, Canada

**Keywords:** interferon, type 1 interferon, STING, age-related, pediatric, viral infection, SARS-CoV-2, COVID-19

## Abstract

Type 1 interferons (T1IFNs) are typically expressed in low concentrations under homeostatic conditions, but upon pathogenic insult or perturbation of the pathway, these critical immune signaling molecules can become either protectors from or drivers of pathology. While essential for initiating antiviral defense and modulating inflammation, dysregulation of T1IFN signaling can contribute to immunopathology, making it and its associated pathways prime targets for immune evasion and disruption by pathogens. This review focuses on the changes in T1IFN signaling across the lifespan, with particular emphasis on the role of the Stimulator of Interferon Genes (STING) pathway in autoimmune and infectious disease susceptibility, especially in the context of viral infections. Aging is associated with diminished T1IFN responsiveness, partially resulting from chronic stimulation of the STING pathway, which contributes to increased susceptibility and impaired viral clearance. Conversely, neonates and young children also show increased vulnerability to certain viral infections, but whether this is driven by T1IFN differences or another mechanism remains incompletely understood. Despite growing interest in T1IFN-based immunotherapies, pediatric and elderly populations remain underrepresented in clinical trials. Here, we advocate for a deeper molecular and systems understanding of how the interferon response evolves across the human lifespan, to inform age-tailored therapeutic approaches and more inclusive study designs, thereby improving outcomes in both the youngest and oldest patients.

## 1 Introduction

Interferons (IFN) are a class of cytokines that ‘interfere’ with viral replication. There are currently three known IFN families, each defined by its use of distinct signaling receptors. Type 1 Interferons (T1IFN) were the first to be characterized and are key effectors and modulators of both innate and adaptive immunity, with broad effects (1). T1IFNs are highly conserved throughout mammalian evolution, and the human genome contains 17 functional genes encoding 16 proteins, including tissue-specific IFN-ϵ and -κ, the ubiquitous IFN-β, and 12 IFN-α subtypes (2).

T1IFN and their downstream Interferon Stimulated Genes (ISGs) play a unique role in controlling inflammation with both pro- and anti-inflammatory effects. This dual nature makes them highly relevant not just in pathogen-mediated disease, but also in autoimmune and autoinflammatory conditions (3). Due to their broad biological activity, dysregulation of the T1IFN pathway can have harmful consequences (4, 5). Given the diverse and numerous roles of T1IFN in immune function, comprehending these is essential to enhance treatment strategies and elucidate underlying pathologies during infections. Furthermore, emerging evidence suggests that the T1IFN response varies across life stages, potentially influencing disease susceptibility, severity, and treatment in both the young and the elderly. In this review, we outline key age-related differences in T1IFN signaling, with particular focus on antiviral responses mediated by the cyclic GMP-AMP synthase (cGAS) – Stimulator of Interferon Genes (STING) pathway, underscoring the need for a deeper understanding of age-specific immunity to improve infection outcomes.

## 2 Type 1 interferon signaling in health and disease

T1IFNs are constitutively expressed at very low concentrations in healthy individuals and are essential for immune homeostasis (6). This basal signaling contributes to the development and maintenance of the hematopoietic system, supporting the proliferation and differentiation of immune cells under steady-state conditions (7, 8). Additionally, low-level T1IFN activity also facilitates immune surveillance, regulates tissue integrity, and primes host defenses in the absence of infection (9, 10). However, dysregulation of T1IFN signaling, through genetic factors or triggered by endogenous stimuli, leads to chronic, sterile inflammation. This activation is a hallmark of several autoinflammatory and autoimmune conditions, including systemic lupus erythematosus (SLE), Aicardi-Goutières syndrome, and STING-associated vasculopathy with onset in infancy (SAVI), where disrupted IFN signaling drives pathological immune activation and tissue damage (3, 11, 12). During a pathogenic insult, T1IFNs transition from homeostatic regulators to powerful antimicrobial agents.

**Figure 1:**
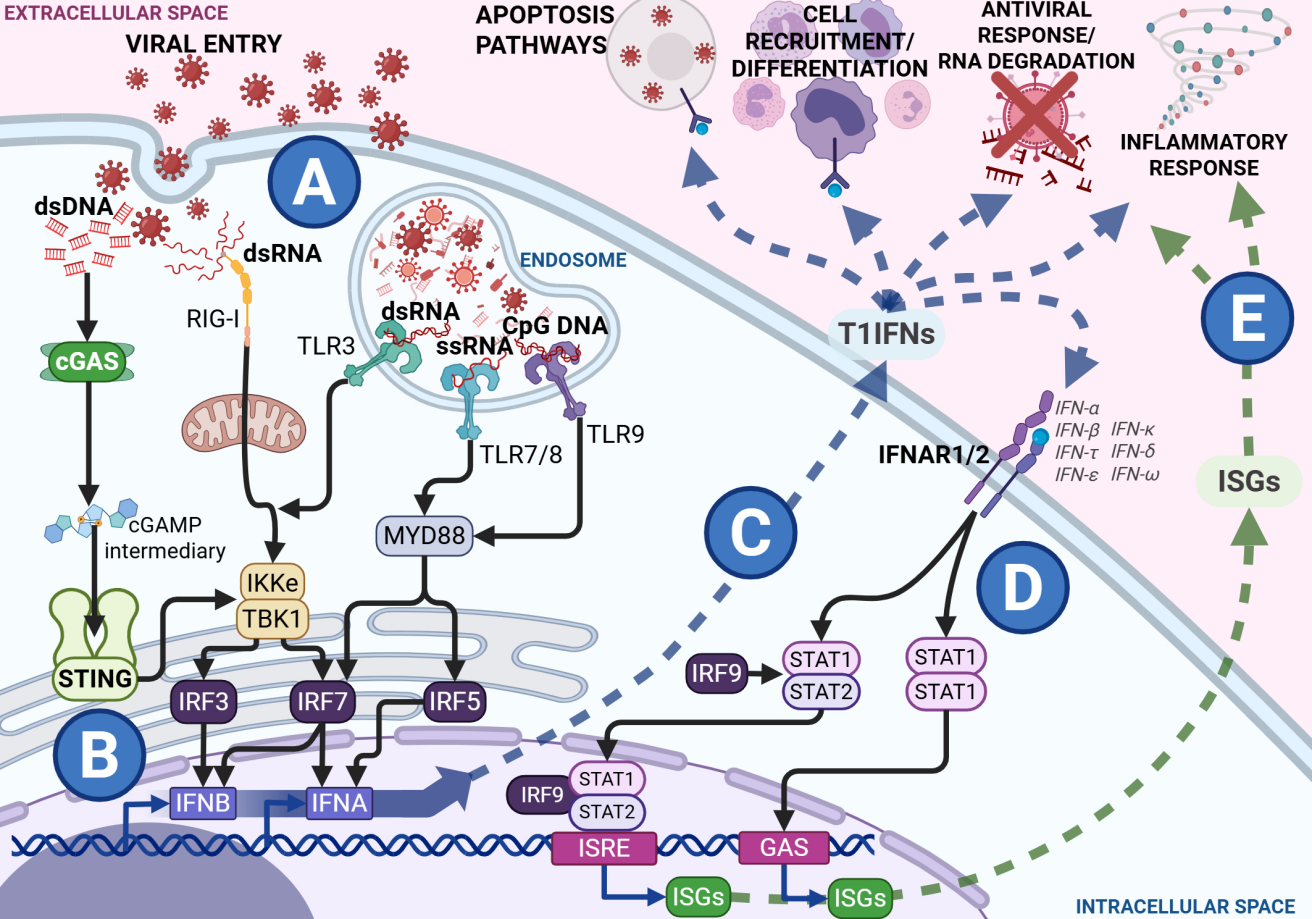
Type 1 interferon (T1IFN) signaling during viral infection with black arrows showing signal transduction within the cytosol from Pathogen Recognition Receptors (PRRs) and the Interferon Alpha Receptor (IFNAR) heterodimer complex to the transcription factors and response elements, the blue arrow showing the release and direct effects of T1IFNs and the green pathway showing the release and effects of Interferon Stimulated Genes (ISGs). During a viral infection and viral entry, viral components are detected through various PRRs in the cytosol and the endosome **(A)**. Cytosolic sensing works through the Stimulator of Interferon Genes (STING) and Retinoic acid-Inducible Gene I (RIG-I) pathways, while endosomal sensing functions involve several Toll-Like Receptor (TLR) pathways. These signal through the endoplasmic reticulum to release Interferon Regulatory Factors (IRFs). Binding of IRFs to promoter regions induces the transcription of T1IFNs, such as IFNA and IFNB **(B)**. These T1IFNs are then released into the extracellular space where they exert antiviral effects such as signaling infected cells to die, recruitment and differentiation of immune cells, RNA degradation, and control of inflammatory response **(C)**. All T1IFNs signal through the same IFNAR heterodimer complex, initiating a Signal Transducer and Activator of Transcription (STAT) cascade, ultimately resulting in transcription of various Interferon ISGs through Interferon-Sensitive Response Elements (ISRE) and Gamma-Activated Sites (GAS) promoter regions **(D)**. These ISGs are released into the extracellular space alongside the T1IFNs, further perpetuating the antiviral and inflammatory responses **(E)**. Figures created in BioRender. Hartnell, L. (2025) https://BioRender.com/ua8nw9b.

During infection, T1IFNs are canonically induced by several pathogen recognition receptors across cellular and tissue compartments in response to diverse stimuli such as bacterial lipopolysaccharide and virion proteins, and intracellular and extracellular detection of nucleotides (**Figure 1A**) (13–16). The STING pathway is a dedicated induction pathway for T1IFN, primarily activated by cytosolic DNA and double-stranded RNA (dsRNA) via the cGAS-Cyclic guanosine monophosphate-adenosine monophosphate (cGAMP) complex (9). Binding of the cGAS sensor to free nucleotides in the cytoplasm produces the intermediary cGAMP, initiating a complex signaling cascade (as shown in **Figure 1**) that culminates in the production of T1IFNs and ISGs (17–19). Secreted T1IFNs act in both autocrine and paracrine manners through the Interferon Alpha Receptor 1/Interferon Alpha Receptor 2 (IFNAR1/2) complex, leading to the production of ISGs (**Figure 1D**). These ISGs contribute to diverse effects, including antiviral function, regulation of antigen presentation, and metabolic modulation (20–24). With their vital role in host defense, T1IFNs are delicately balanced so as to avoid the immunopathology that comes with dysregulated signaling. This duality is evident during infection, where the nature, timing, and context of T1IFN signaling shape the course and outcome of disease.

## 3 Type 1 interferons during infection

The immunological impact of T1IFNs during infection is defined not only by their induction pathways but also by pathogen-specific evasion strategies. Signaling through cGAS-STING has been shown to be essential for T1IFN responses to DNA-containing pathogens, with genetic disruption altogether preventing such responses to bacteria (14), as well as DNA viruses and retroviruses. Bacterial induction of cGAS-STING-IFN is not fully elucidated, as, unlike viruses, successful bacteria do not release their genetic material into the cytoplasm of host cells. However, it has been proposed that bacterial induction of T1IFN relies on mistakes in bacterial infection processes, such as the accidental secretion of genetic material or bacterial cyclic dinucleotides (CDNs) along with toxins (25).

The consequences of T1IFN induction in bacterial infection are context-dependent and can be paradoxical (26). While T1IFNs induce bacterial autophagy by host dendritic cells, they also increase cell motility, facilitating cell-to-cell spread and bacterial escape (27). In contrast, during viral infection, the cGAS-STING pathway quickly senses viral nucleotides released into the cytoplasm during the infection process, inducing T1IFN and its downstream effectors (as seen in **Figure 1**). Interference in viral infection by ISGs occurs at most stages of the viral replication cycle, including preventing viral entry, protein and mRNA synthesis, and assembly (28). Additionally, IFNs and ISGs contribute to wider immune defense by mediating cell-cell interactions and recruitment via altering cell receptor and chemoattractant expression and promoting antigen presentation (**Figures 1C, E**) (28–30). The timing, magnitude, and duration of the T1IFN response is critical for optimal function in host defense. T1IFN signaling is highly beneficial in the host response mechanisms to acute viral infection but becomes detrimental in prolonged or chronic infection, such as in human immunodeficiency virus (HIV) infection, where it induces a negative feedback loop that reduces the immune response over time, promoting a proviral state (30–32). This exemplifies the finely tuned balance of STING-IFN signaling that is integral to the proper functioning of immune responses, and how pathogens can utilize its mechanisms to perpetuate infection.

## 4 Evasion and disruption of STING-IFN signaling by pathogens

Given the importance of T1IFNs to immune response and pathogen clearance, many infectious agents disrupt related pathways to either evade detection or improve escape (33, 34). These various strategies not only perpetuate infectious agents within the body but also increase immune-mediated tissue damage (35, 36). Many bacteria induce T1IFNs, especially intracellular bacteria, like Listeria, which stimulate the cGAS-STING pathway (14). Bacterial strategies for the disruption of T1IFN responses typically rely on taking advantage of inflammation and IFN-induced apoptosis of essential immune effector cells (3, 37).

Numerous viruses express viral proteins that prevent signal transduction through the STING-IFN pathway, such as the papain-like protease of coronaviruses (38–40). Non-structural proteins in Respiratory Syncytial Virus (RSV) suppress T1IFN responses, and higher T1IFN levels are associated with better outcomes from the infection (41). Beyond the damage caused by invading microbes, the host’s response to pathogens can lead to hyperinflammation and other pathologies with severe outcomes. Disrupting or delaying the T1IFN response can result in a poorly timed influx of inflammatory cells that perpetuates pathological inflammation and a pro-viral state that is unable to resolve successfully (3, 42, 43). Therefore, understanding the dynamic interplay between pathogens and the host’s immune response is crucial to improving the understanding of infection progression and outcomes.

## 5 Type 1 interferon signaling in early life

Compared to their adult counterparts, the current understanding of the T1IFN response to infection in early life is limited. In 2019, infectious diseases accounted for up to 49% of deaths in children under five years of age worldwide, with lower respiratory infections responsible for 13.9% of these deaths (44). The immune systems of neonates and children rely heavily on innate immunity due to the naïveté of the adaptive system (45–48). Infant immunity also relies on vertically transmitted antibodies and antimicrobials through their mother’s placenta and breastmilk. Nevertheless, these protective substances wane six months post-birth for placental antibodies and diminish with the cessation of breastfeeding for other immune factors (49–51).

**Figure 2:**
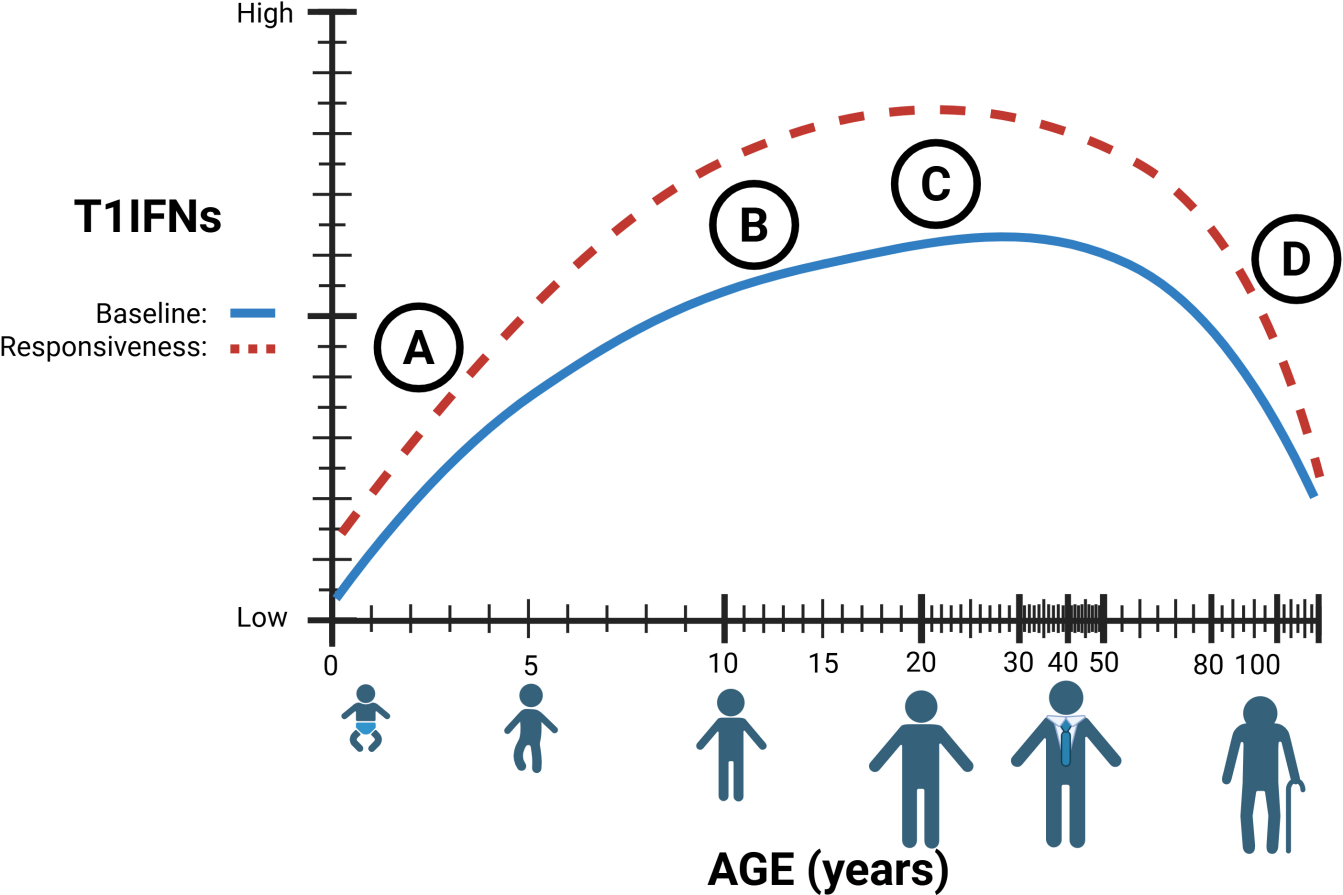
**(A)** Type 1 interferon (T1IFN) signaling in early life is believed to be diminished resulting from immature and decreased numbers of immune cells. **(B)** T1IFN signaling increases after puberty, with hormones significantly affecting T1IFN signaling pathways. **(C)** The true immunological ‘peak’ of T1IFN is still unknown. **(D)** Lower baseline T1IFNs through chronic STING stimulation, combined with lower circulating T1IFN-producing cells results in decreased sensitivity and responsiveness in elderly individuals. Figures created in BioRender. Hartnell, L. (2025) https://BioRender.com/x7l3f99.

Particularly in neonates, numerous aspects of immune responses appear counterintuitive to infection clearance. Immune cell populations in children differ from those in adults and change dynamically as individuals age, impacting both the capacity and coordination of immune response, with implications for systemic immunity (52–54). At the cellular level, neonatal innate cells produce higher quantities of single cytokines such as interleukins IL-1β, IL-6, IL-23, and IL-10 compared to adults but have diminished simultaneous multi-cytokine responses (55, 56). While almost all immune cell types produce T1IFN, plasmacytoid dendritic cells (pDCs) are the major drivers of T1IFN production during infection (57–60). These specialized dendritic cells have the capacity to produce substantial amounts of T1IFN rapidly and can infiltrate other tissue types such as the airway mucosa (61). Their potent T1IFN production and ability to migrate into infected tissue make them crucial to host immune responses despite representing a small fraction of peripheral blood mononuclear cells (PBMC) (62, 63). Neonatal mononuclear cells produce significantly lower levels of IFN-α than adults, which is less effective at activating pDCs, and while production increases after two months, it remains lower until about 18 months of age (**Table 1**) (64, 65). Multi-omics profiling of infant PBMCs reveals an elevated baseline of antiviral ISGs, suggesting a partially primed innate state (66). *Ex vivo* studies of cord blood pDCs also show decreased activity of interferon regulatory factors IRF3 and IRF7, with reduced IRF3 activation observed in cord blood mononuclear cells, and decreased production of the intermediary cGAMP molecule (**Figure 2A**) (67–69). IRF3 is a key regulator in IFN-β production and Transforming Growth Factor β (TGF-β), by preventing IRF3 phosphorylation and subsequent nuclear translocation, inhibits IFN-β transcription (70–72). Interestingly, Okamoto et al. found that pediatric populations have higher levels of circulating TGF, suggesting one possible mechanism for decreased childhood T1IFN responses (73). These differences in innate T1IFN responses may contribute to observed differences in susceptibility to and severity of infection in children (**Table 1**).

**Table 1:** Age-related differences in type I interferon (T1IFN) subtypes.

T1IFN subtype	Early life	Adult/aged life
IFN-α	Reduced production in neonates; low until ~18 months (64, 67–69).	Diminished PBMC responses to viral/PRR stimulation (108).
IFN-β	Impaired IRF3 activity; TGF-β inhibition of transcription (70–73).	Basal transcripts decline with age (109).
IFN-ϵ	Data lacking in neonates/infants.	Basal transcripts decline with age (109).
IFN-ω	Data lacking.	Basal transcripts decline with age (109).
IFN-κ/others	Limited data; infants show elevated baseline ISGs (66).	Sparse evidence; some subtypes are reduced (108, 109).

Early life is characterized by reduced IFN-α and IFN-β production, with limited data available for other subtypes. In elderly populations, basal transcript levels of several IFNs decline and PBMC responsiveness is diminished, underscoring the need for further age-specific characterization.

Children exhibit higher infection rates compared to adults, particularly with respiratory viruses, alongside increased rates of coinfection; however, most children experience more favorable health outcomes (74–77). This may be attributed to differences in first-line defense involving T1IFNs. Compared to adults, neonatal and infant infection with RSV is associated with less circulating pDCs, reduced T1IFN responses, and Retinoic acid-Inducible Gene I (RIG-I) signaling (41). More severe RSV infections in infants correlate with lower nasal IFN levels, bronchiolitis, and respiratory failure (78). Contrary to RSV responses, children have a stronger innate immune response to influenza than adults, driven by increased T1IFNs (79). In children, impaired IRF production and T1IFN signaling during influenza infection are associated with susceptibility, and increased T1IFN appears to be protective from severe disease (80, 81). Similar to influenza, pediatric infections with Severe Acute Respiratory Syndrome Coronavirus 2 (SARS-CoV-2) are associated with stronger innate IFN responses than adults (82, 83). Children, particularly in the upper airway mucosa, exhibit higher pre-infection levels of T1IFN, allowing for more efficient early viral control and limiting systemic spread (84, 85). Additionally, *in vitro* co-culture of pediatric nasal epithelial and immune cells shows enhanced epithelial-immune crosstalk, resulting in more rapid and higher magnitude T1IFN induction than adults (86). In early life, T-cells are generally less polyfunctional, producing fewer cytokines simultaneously (53). Polyfunctionality of T cells can influence the course of SARS-CoV-2 infection and, in children, the presence of polyfunctional SARS-CoV-2-specific CD4^+^ T cells correlates with seroconversion, suggesting a role in the development of lasting immunity (87, 88). This apparent divergence in adult and pediatric responses underscores that the two immune systems function differently. Understanding these differences is crucial for enhancing prevention strategies, therapeutic interventions, and overall health outcomes.

## 6 Therapeutic use of type 1 interferon in early life

The immune system develops at varying rates from infancy to old age and relies heavily on the accumulation of immunity through repeated exposures. As a result, there is no defined age at which an individual is considered immunologically “mature” (**Figure 2**) (52). This highlights a need for age-specific treatment approaches and a deeper understanding of the baseline immune environment in children.

Clinical trials of T1IFNs therapy in adults for SARS-CoV-2 infection have shown significant success in reducing viral load, disease severity, and transmission, particularly when administered before peak viral load (89–92). While most trials exclude both young children and the elderly, some studies have demonstrated the effectiveness of T1IFN in reducing viral load in pediatric patients (93). Both IFN-α and IFN-β have been proven safe and effective for treating chronic viral infections in children; however, further research is needed before they can be widely applied for acute infections (94, 95). Given the critical role of timing in IFN therapy, more research is necessary to understand differences in viral response kinetics between adults and children.

## 7 Type 1 interferon signaling in adult life

Given the role of STING in sensing and responding to damaged DNA, it is highly relevant in understanding health and disease in aging populations. Aging increases the risk and prevalence of cellular and genomic damage (96). Accumulated damage results in the release of self-DNA into the cytoplasm and stimulation of the STING-IFN pathway (97), which has been implicated in age-related chronic inflammation, immunosenescence, and cancer (98, 99). These factors, combined with differences in circulating immune cell populations, lead to an increased susceptibility of aged individuals to disease (100–103). Notably, the T1IFN-producing pDCs decrease in the elderly, and their capacity to produce T1IFNs diminishes, reducing their capacity to mount a response (**Figure 2**) (100, 104, 105). Additionally, key pathogen recognition pathways that induce T1IFN are decreased in later life, such as RIG-I and numerous TLR pathways, across multiple cell types (106, 107). In short, elderly populations exhibit chronic STING activation, leading to reduced T1IFN production and sensitivity, which results in dysregulated cell differentiation and recruitment during immune responses to infection.

While T1IFNs (including IFN-α, IFN-β, IFN-ϵ, and IFN-λ) are critical for antiviral immunity, the extent to which they change with age varies by subtype, and such characterization remains incomplete (as summarized in **Table 1**). *Ex vivo* stimulation of PBMCs from older adults shows delayed and diminished production of IFN-α in response to PRR agonists, including those targeting STING and RIG-I pathways (108). Transcriptomic analysis of upper from SARS-CoV-2-negative individuals shows that basal mRNA transcripts of IFN-ϵ and IFN-λ decline with increasing age, in addition to IFN-α and IFN-β, although to a lesser extent (**Figure 2D**) (109). Notably, evidence for IFN-κ and other subtypes in the aging immune system remains sparse, highlighting a critical knowledge gap and an area for future investigation across all ages (**Table 1**).

Deficiencies in T1IFN signaling in aged individuals have been associated with poorer vaccine responses and increased viral load during infection (110, 111). This combination of factors contributes to an overarching susceptibility to viral infections during later life and poorer outcomes from these infections (112). Repeated and chronic infections in elderly populations exacerbate this vulnerability, resulting in a functionally exhausted pDC population that is less able to respond to T1IFN stimulation (113). Among these, human cytomegalovirus (HCMV) is particularly influential. Lifelong HCMV latency is characterized by sustained immune stimulation, chronic STING pathway activation, and dysfunctional memory T cells that can accelerate immunosenescence and impair T1IFN responsiveness (114). The cumulative burden of chronic infections such as HCMV not only reshapes immune cell compartments but also leaves older adults less able to respond effectively to acute viral threats.

These differences in immunity may help to explain disproportionate susceptibility and severity of numerous viruses. RSV is typically more severe in both the young and the elderly, partly because it suppresses T1IFN responses (41, 115). In the elderly, poor RSV outcomes result from multiple factors, including reduced IFN sensitivity, immune exhaustion, and defective immune memory, culminating in an inability to produce a sufficient response to viral assault (116, 117). In geriatric influenza infections, PBMC-derived production of IFN-α is decreased, but the individual capacity of pDCs to produce the cytokine remains intact (118). However, localized expression of ISGs in geriatric airway epithelial cells is skewed to favor an inflammatory phenotype, which impairs viral clearance (119). In addition to RSV and influenza, older adults are also more likely to have poorer outcomes of SARS-CoV-2 infection than their younger counterparts (120). Disease severity of SARS-CoV-2 has been linked to the timing and magnitude of the T1IFN response (84, 121, 122). Aging-related dysfunction of T1IFN, and its role in inflammation and immunosenescence, contributes to increased risk of cytokine storm and adverse outcomes of SARS-CoV-2 infection (123, 124). Taken together, STING-mediated chronic inflammation, lower populations of T1IFN-producing cells, and reduced T1IFN sensitivity become a potentially disastrous combination for older adults with viral infections. To tackle this, intervention strategies that modulate activation along the STING-T1IFN pathway present promising avenues for reversing immunosenescence and restoring T1IFN responsiveness in elderly populations (125, 126). This rationale underpins the growing interest in T1IFN-based therapies in adult and elderly populations.

## 8 Therapeutic use of type I interferon in adult life

Given its inherent antiviral properties, T1IFN has the potential to complement standard treatments for viral infections. IFN-β is already an established and effective therapy for reducing relapse frequency and disease progression in multiple sclerosis (127). Additionally, IFNs and related modulators are widely used as both frontline and alternative therapies for viral infections, in acute and chronic stages, often in combination with standard care (94, 128–132). A 2022 scoping review on interferon-based therapies for human respiratory viral infections found that 66% of trials evaluated IFN-α as the primary intervention, with rhinovirus (40% of trials) and SARS-CoV-2 (29% of trials) being the most studied pathogens (133). Elderly populations, however, have historically been underrepresented in clinical trials involving IFN-based treatments. Older participants are also more likely to experience higher rates of adverse events and study withdrawal compared to younger participants (134). Despite these challenges, IFN treatment remains a promising option for novel viral infections such as SARS-CoV-2, particularly in elderly populations, where age is a significant risk factor for severe outcomes (135). Given the observed age-related differences in immune response and infection susceptibility, it is essential to ensure broader representation across all age groups in clinical trials, including individuals at both ends of the age spectrum.

## 9 Discussion

Interferon signaling during infection differs between pediatric, adult, and geriatric populations, influencing disease susceptibility as well as progression to morbidity and mortality. At one end of the lifespan, the early-life environment balances the cost-benefit of growth processes against the need to fight infections. In neonates, immune cells are less polyfunctional, and interferon production is diminished throughout the first 18 months of life. In contrast, children have stronger innate T1IFN responses, resulting in better outcomes than adults despite experiencing higher infection rates. These patterns are observed in both common and severe viral infections. At the other end of the spectrum, the aging immune system is characterized and driven by chronic inflammation, diminished cell populations, and immune exhaustion. Within this context, chronic stimulation of the STING pathway, resulting from accumulated genome damage, along with decreased T1IFN production and sensitivity, perpetuates susceptibility and poor outcomes to disease in the elderly, particularly in cases of respiratory virus infection.

In a world increasingly at risk from novel pathogens such as SARS-CoV-2, T1IFNs offer a valuable treatment option due to their dual role in regulating innate immune responses and exerting antiviral effects. T1IFNs not only restrict pathogen replication but also modulate immune responses to mitigate or prevent the immunopathology associated with severe infections. However, applying this therapy across age groups requires a deeper understanding of age-related differences in T1IFN pathway function and regulation.

This review has outlined age-related differences in the T1IFN pathway, regulated by the cGAS-STING pathway, and response to viral infection (as summarized in **Tables 1** and **2**). Although significant progress has been made in the development of T1IFN-based therapies, few studies have included or considered pediatric populations. Identifying age-specific T1IFN response characteristics, kinetics, and magnitude shift could help optimize treatment protocols, improving infection management and reducing treatment burdens. Additionally, a deeper understanding of the developing innate interferon response could inform targeted and age-specific treatments for both pediatric and geriatric viral infections, such as RSV, and provide a basis for host-focused therapies to combat future novel pathogens. Ultimately, age-related differences in the innate immune response warrant greater attention, including explicit consideration in the design of clinical trials.

**Table 2:** Comparison of type I interferon (T1IFN) signaling in early life and adult/aged life.

Aspect	Early life	Adult/aged life	Therapeutic implications
BASELINE IMMUNITY	Reliance on maternal antibody protection; innate immunity dominates (45–51).	Chronic STING activation, immunosenescence, inflammation (96–99).	Tailor therapies to immature vs. exhausted immunity.
pDCs	Reduced numbers and IFN-α output until ~18 months (64, 67–69).	Decline in frequency and function with age (100, 104, 105).	Boost pDC responses in both groups.
T1IFN PRODUCTION	Lower IFN-α, impaired IRF3/7, higher TGF-β inhibition (67–73).	Reduced IFN-α/β/ϵ/λ; impaired responsiveness; chronic infection burden (108, 109, 114).	Enhance IFN in neonates; restore responsiveness in elderly.
VIRAL OUTCOMES	RSV: weak IFN response, severe disease (41, 78). Influenza & SARS-CoV-2: stronger IFN, better control (79–83, 85).	RSV: poor IFN sensitivity, severe disease (41, 115–117). Influenza & SARS-CoV-2: delayed/dysregulated IFN, worse outcomes (84, 118–124).	Age-specific IFN therapy may mitigate severity; timing critical.
T CELL FUNCTION	Reduced polyfunctionality; protective polyfunctional CD4+ T cells in SARS-CoV-2 (53, 87, 88).	Impaired memory from chronic infections, poor vaccine responses (100–113).	Support T cell polyfunctionality in young; reverse exhaustion in elderly.
T1IFN THERAPIES	IFN-α/β safe in chronic infections; limited data in acute (93–95).	IFN widely used (e.g., MS, viral infections); elderly underrepresented, more adverse events (94, 127–135).	Include both age groups in trials; early dosing improves outcomes (89–92).

Key differences are summarized across baseline immunity, plasmacytoid dendritic cells (pDCs), interferon production, infection outcomes, T cell function, and therapeutic use. This highlights how age-specific immune features shape susceptibility to infection and responsiveness to T1IFN-based therapies, underscoring the need for tailored treatment strategies and inclusive clinical trial designs.

## 10 Future directions

Together, these findings underscore the importance of contextualizing T1IFN biology across the lifespan. Building on this foundation, future work should leverage emerging technologies and therapeutic strategies to address these age-related differences. Advancing our understanding of T1IFN biology will require integrative approaches that go beyond single-dimensional measurements of cytokine production. The emergence of multi-omics profiling i.e., combining transcriptomics, proteomics, metabolomics, and epigenomics, offers the potential to characterize age-specific signatures of STING activation and T1IFN responsiveness at a higher resolution than ever before. Combining this with systems-level computational modelling could identify predictive biomarkers of infection trajectory, antiviral protection, or immunopathology, as well as regulatory nodes that can be leveraged for therapeutic intervention. In parallel, there is an urgent need to expand translational studies into underrepresented pediatric and geriatric populations. Age-tailored modulation of the STING-T1IFN axis may help restore responsiveness in the elderly or temper hyperresponsiveness in the neonate, ultimately reducing infection-related morbidity at both ends of the lifespan. Incorporating these perspectives into clinical trial design will not only refine therapeutic strategies for existing pathogens but also enhance preparedness against future viral threats.
